# High Dose of Acute Normobaric Hypoxia Does Not Adversely Affect Sprint Interval Training, Cognitive Performance and Heart Rate Variability in Males and Females

**DOI:** 10.3390/biology11101463

**Published:** 2022-10-06

**Authors:** Raci Karayigit, Rodrigo Ramirez-Campillo, Burak Caglar Yasli, Tomasz Gabrys, Daniela Benesova, Ozcan Esen

**Affiliations:** 1 Faculty of Sport Sciences, Ankara University, Gölbaşı, Ankara 06830, Turkey; 2 School of Physical Therapy, Faculty of Rehabilitation Sciences, Exercise and Rehabilitation Sciences Institute, Universidad Andres Bello, Santiago 7591538, Chile; 3 Department of Physical Education and Sports, Iğdır University, Iğdır 76000, Turkey; 4 Department of Physical Education and Sport, Faculty of Education, University of West Bohemia, 30100 Pilsen, Czech Republic; 5 Department of Sport, Exercise and Rehabilitation, Northumbria University, Newcastle-upon-Tyne NE1 8ST, UK

**Keywords:** altitude, high-intensity interval training, human physical conditioning, sex

## Abstract

**Simple Summary:**

Sprint interval training (SIT) is a feasible and time-efficient alternative to classical endurance training that has gained popularity among athletes because of its ability to elicit physiological and cardiorespiratory adaptations in a shorter amount of time than traditional endurance training. Further, popular altitude/hypoxic training techniques include intermittent hypoxic training, in which athletes exercise at submaximal levels under simulated hypoxia while living at sea level (normoxia). Hypoxic exercise is likely a more potent stimulant to upregulate muscle factors (e.g., mitochondrial biogenesis, oxidative, and glycolytic enzymes) than similar normoxic exercise. However, SIT in hypoxia may disturb acute performance indices during sprint intervals. Hypoxia may also impair cognitive function. Acute hypoxia may decrease cognitive performance in areas such as memory and executive functioning. Moreover, males and females may have distinct athletic performance responses to SIT and hypoxia. However, to date, there is no study that has investigated the effects of different doses of acute normobaric hypoxia on SIT and cognitive performance, nor has there been research investigating potential sex-based differences.

**Abstract:**

Although preliminary studies suggested sex-related differences in physiological responses to hypoxia, the effects of sex on sprint interval training (SIT) performance in different degrees of hypoxia are largely lacking. The aim of this study was to examine the acute effect of different doses of normobaric hypoxia on SIT performance as well as heart rate variability (HRV) and cognitive performance (CP) in amateur-trained team sport players by comparing potential sex differences. In a randomized, double-blind, crossover design, 26 (13 females) amateur team-sport (football, basketball, handball, rugby) players completed acute SIT (6 × 15 s all-out sprints, separated with 2 min active recovery, against a load equivalent to 9% of body weight) on a cycle ergometer, in one of four conditions: (I) normoxia without a mask (F_i_O_2_: 20.9%) (CON); (II) normoxia with a mask (F_i_O_2_: 20.9%) (NOR); (III) moderate hypoxia (F_i_O_2_: 15.4%) with mask (MHYP); and (IV) high hypoxia (F_i_O_2_: 13.4%) with mask (HHYP). Peak (PPO) and mean power output (MPO), HRV, heart rate (HR), CP, capillary lactate (BLa), and ratings of perceived exertion (RPE) pre- and post-SIT were compared between CON, NOR, MHYP and HHYP. There were no significant differences found between trials for PPO (*p* = 0.55), MPO (*p* = 0.44), RPE (*p* = 0.39), HR (*p* = 0.49), HRV (*p* > 0.05) and CP (response accuracy: *p* = 0.92; reaction time: *p* = 0.24). The changes in MP, PP, RPE, HR, CP and HRV were similar between men and women (all *p* > 0.05). While BLa was similar (*p* = 0.10) between MHYP and HHYP trials, it was greater compared to CON (*p* = 0.01) and NOR (*p* = 0.01), without a sex-effect. In conclusion, compared to normoxia, hypoxia, and wearing a mask, have no effect on SIT acute responses (other than lactate), including PP, MP, RPE, CP, HR, and cardiac autonomic modulation either in men or women.

## 1. Introduction

Compared to traditional endurance training, sprint interval training (SIT) is a viable and time-efficient alternative to induce physiological and cardiorespiratory adaptations having become popular among athletes [1]. Indeed, SIT has been reported to improve mitochondrial enzyme activity [2], reduce glycogen utilization and lactate accumulation during work-matched exercise [3], enhance aerobic capacity and performance [4], and improve muscle buffering capacity [5]. Moreover, it is known that hypoxic training can induce upregulation of mitochondrial biogenesis [6,7], oxidative and glycolytic enzymes [6,8,9], and monocarboxylate transporters [10]. Therefore, the application of SIT in altitude/hypoxic environments to enhance endurance and high-intensity exercise performance has recently received unprecedented interest from athletes [11], although it still remains to be explored further. 

The SIT in the hypoxia method is of interest in a wide range of team sports as well as endurance sports [12] as it demands remarkably high recruitment of type II fibers [13,14]. Besides its efficacy being dependent on both duration and the sprint:rest ratio, the severity of hypoxia would be also a crucial factor that can affect the aforementioned adaptations. Indeed, Bowtell et al. [15] have reported the highest changes in physiological parameters and performance at F_i_O_2_: 12% between four different hypoxic conditions (F_i_O_2_: 12–15%). Further studies have also shown [16,17] that the higher decrease in sprint performance occurred at the higher doses of hypoxia (F_i_O_2_: 12.5% vs. F_i_O_2_: 14.5%). However, Kon et al. [18] have reported that there is no difference in capillary blood lactate (BLa) concentration and sprint performance between normoxic (F_i_O_2_: 21%), and two different degrees of hypoxia conditions (F_i_O_2_: 16.4% and F_i_O_2_: 13.6%). Additionally, it has been reported that improvements in VO_2_, time to exhaustion and ratings of perceived exertion (RPE) after a 4-week of SIT were similar between hypoxia (F_i_O_2_: 13.6%) and normoxia [19], suggesting that the hypoxic degree might be too low to induce more training responses. Therefore, further studies are needed to improve the understanding of the effect of degrees of hypoxia on SIT performance, which may provide positive implications for the programming of the training intervention. 

Whilst previous studies have mostly investigated the effect of SIT on metabolic parameters, such as VO_2_, oxidative enzymes, and mitochondrial biogenesis, its impact on heart rate variability (HRV) and cognitive performance (CP) under hypoxic conditions is poorly understood. The HRV is a non-invasive indicator of cardiac activity, with high HRV indicating healthy cardiac activity [20]. The activity of the cardiac autonomic system (CAS) has been reported to decrease at rest in hypoxia [20] compared to normoxia, but both CAS and sympathetic activity seems to be maintained during exercise in hypoxia compared to normoxia [21,22]. Although these data indirectly suggest that hypoxic condition has the potential to influence HRV, empirical evidence to support this, particularly in different degrees of hypoxia, is presently lacking.

Hypoxia can also affect cognitive performance (CP) [23]. Acute hypoxic exposure may reduce CP in domains such as memory and executive functions [24]. Moreover, the use of masks is common during hypoxia simulation; however, the mask may increase breathing difficulty for athletes, creating a psychological strain [25], with a negative psychological effect [26]. However, how CP is affected during SIT under different doses of hypoxia (i.e., high vs. moderate hypoxia) is unknown. Clarification of such a research gap would help to understand the inter-relationships between hypoxia dose and SIT programming variables. 

The effects of sex-based differences on SIT and hypoxia are also of interest in a variety of sports, as males and females may manifest different athletic performance responses [27]. Females, compared to males, have been reported to have greater fatigue resistance and better recovery, despite higher cardiovascular strain and RPE in during 8 × 30 m all-out sprints [28] and 6 × 4 min high-intensity interval training bouts in normoxia [29]. Females were also found to have lower BLa concentration during hypoxic exercise (F_i_O_2_: 13%) and higher glucose levels during recovery when compared to males [30]. These observations may be partially based on the finding that sex-dependent differences in intermittent exercise occur during rest intervals since females have been reported to present faster adenosine triphosphate (ATP) recovery [31]. However, if such sex-based differences in SIT performance as well as HRV and CP under different doses of hypoxia (i.e., high vs. moderate) exist remains to be elucidated. Therefore, the aim of this study was to examine the acute effects of different doses of normobaric hypoxia on SIT performance as well as HRV and CP in amateur-trained team-sport players by comparing potential sex differences. Our hypothesis is that hypoxia would affect SIT performance to a greater degree in males compared to females. We also hypothesized that these effects would be greater in a higher dose of hypoxic conditions. 

## 2. Materials and Methods

### 2.1. Participants

Thirteen female (age 21 ± 1 years; height 168.0 ± 7.7 cm; body mass 60.7 ± 5.8 kg; VO_2__max_ 50.0 ± 2.1 mL/kg/min; mean ± SD) and thirteen male (age 22 ± 1 years; height 178.2 ± 8.6 cm; body mass 76.3 ± 3.7 kg; VO_2__max_ 54.3 ± 3.4 mL/kg/min) amateur-trained team sport players participated in this study. Participants had, currently or in the previous 3 months, no musculoskeletal injury. Exclusion criteria were pre-existing acute or chronic diseases, having exposure to a real or simulated altitude higher than 1500 m during the previous 3 months., regular smoking, and any travel to altitudes > 2000 m within the 3 months preceding this study. All participants had completed at least 5 h weekly all-out sprint type activities. 

An incremental cycling protocol up to exhaustion was performed to determine the VO_2__max_ [32]. The participants were informed about the experimental details, and they gave written informed consent before commencing the study. Participants were informed to refrain from vigorous physical activity, and consumption of caffeine, alcohol and ergogenic aid that might improve performance acutely (i.e., nitrate, l-arginine, l-citrulline and bicarbonate) at least 24 h before each trial. Participants were asked to record their 24-h dietary intake before the first trial and to replicate the same diet before the subsequent trials. The testing sessions were carried out in conformity with ethical standards (i.e., updated version of the Declaration of Helsinki). Sinop University, Human Research Ethics Committee approved the study protocols (decision no: 2021/30). 

### 2.2. Study Design

Participants attended the laboratory on five occasions, separated by 72 h. The first visit included the completion of study documentation, baseline anthropomorphic and determination of VO_2max_ via an incremental cycling protocol up to voluntary exhaustion [32]. Participants also completed a familiarization SIT protocol in which data was collected but was only used to display any learning effects and not for further analyses. Following completion of this initial familiarization visit, participants were assigned to perform the SIT protocol either (I) 900 m (F_i_O_2_: 20.9%) without wearing a hypoxia generator mask (CON), (II) 900 m (F_i_O_2_: 20.9%) wearing a hypoxia generator mask (NOR), (III) 2500 m (F_i_O_2_: 15.4%; MHYP) wearing a hypoxia generator mask, or (IV) 3500 m (F_i_O_2_: 13.5%; HHYP) wearing a hypoxia generator mask in a randomized, counter-balanced, double-blind, crossover design. A researcher who was not involved in data collection and analyses conducted the arrangements concerning session order and blinding. Participants were not able to see and/or read any data and/or results during exercise. Further, no data and/or results were shared with participants until they completed all experimental sessions. All sessions were conducted during the luteal phase of the female participants’ menstrual cycle [33,34] 

**Figure 1:**
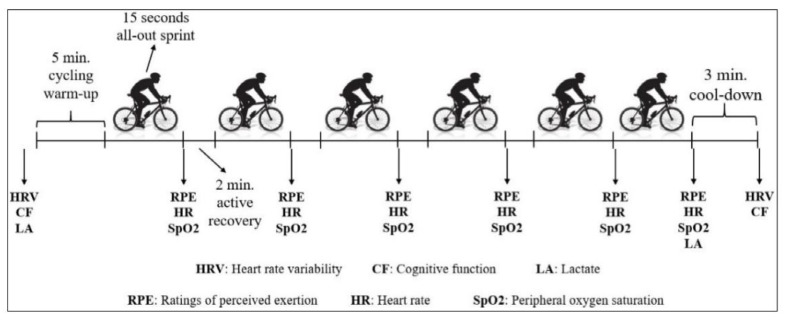
Schematic representation of the sprint interval training protocol.

To simulate the specified altitudes during the SIT sessions, the participants wore a mask connected to the Everest Summit II-Altitude Generator (Hypoxico, NY, USA). To verify hypoxic and normoxic conditions, oxygen was assessed by a pulse oximeter (Hypoxico Oxycon, USA) attached to the participants’ fingers during sprint bouts [7,15,17]. The four SIT trials were carried out at the same time of the day (7:00–9:00 a.m.) at the laboratory in which the room temperature (22 ± 1.2 °C) and humidity (55 ± 5.3%) were controlled. HRV was recorded for 5 min before and after (~1 min post) SIT protocol. Following HRV measurements (~45 s post), CP was also measured for 3 min before and after the SIT protocol. HR (lactate scout, USA), RPE and BLa samples were measured at rest, after each sprint and at the end of the test protocol (Figure 1).

### 2.3. SIT Protocol and Computed Performance Indices

The SIT protocol included a 5 min warm-up (cycling at 60 W), six repeated cycling sprints (total time = 11.5 min), and a 3 min cool- down at 60 rpm and 60 W. The SIT trial was conducted in a cycle ergometer (Monark Exercise AB, Vansbro, Sweden), involving 6 × 15 s all-out sprints, separated with 2 min active recovery at 60 W (60 rpm against 1 kg load), against a load equivalent to 9% of body weight. This SIT protocol improved time to exhaustion and VO_2__max_ in men and women after 3 weeks [1]. During each sprint, peak (PPO) and mean power output (MPO) were computed and recorded (Monark Anaerobic Test Sofware, Version 3.3.0.0., Vansbro, Sweden), as well as HR, RPE and peripheral oxygen saturation (SpO_2_) after each sprint. 

### 2.4. Heart Rate Variability

Upon arrival at the laboratory and following 15 min of rest in the supine position, HRV was recorded for 5 min before the SIT protocol by using validated equipment (Omega Wave 800, OW, Portland, OR, USA) [33]. HRV was also recorded for 5 min ~1 min after completing the SIT protocol. Three of the seven electrodes used during measurements were thoracic Wilson electrodes and four tarsal limb electrodes. Participants were asked to remain silent and still during the measurements while maintaining their routine respiratory rate. Using validated software (Omega Wave Sport Tech, Portland, OR, USA) automatic records were obtained for the following HRV outcomes: standard deviation of normal-to-normal (NN) intervals (SDNN), standard deviation of successive differences (SDSD), root mean square of successive differences (RMSSD; i.e., parasympathetic activity), total power (TP; variations between NN intervals), the ratio of low- and high-frequency powers (LF/HF; i.e., sympatho-vagal balance), high-frequency power (HF; i.e., vagal activity, low-frequency power (LF; i.e., combination of sympathetic and parasympathetic activity) [35,36]. 

### 2.5. Cognitive Performance

Mean response accuracy (%) and response times (ms) were collected as CP outcomes, using a modified version of the Flanker task [37,38], with validated software (Inquisit Lab 5.0). Briefly, the participants looked at a white background (with a yellow star at the centre) were five black arrows suddenly appear (in random order, e.g., < < > < < <; > > < > >; etc.) during 200 ms. Thereafter, the participants had to respond as quickly and accurately as possible regarding the direction of the middle arrow (i.e., either > or <), by pressing a button with their left or right index finger. Participants had up to 2000 ms from the onset of the stimulus to respond. After 20 practice trials, the participants wore earplugs and performed 100 trials, with an inter-trial interval of 1000 to 2000 ms. The total duration of the test was ~3 min. 

### 2.6. Statistics

All data were analyzed using the IBM SPSS statistic software package for Windows, version 22.0 (IBM Corp., Armonk, NY, USA). All dependent variables (PPO, MPO, HRV parameters, CP parameters, RPE, BLa, SpO_2_) were analyzed using a two-way repeated-measures (conditions [CON, NOR, MHYP, HHYP] × times [pre, post] or sprints [1,2,3,4,5,6] x genders [male, female]) analysis of variance (ANOVA). The effect sizes were calculated using partial eta squared (η_p_^2^), derived from the ANOVA, and classified as trivial (<0.10), moderate (0.25–0.39), or large (≥0.40) [39]. If significant interactions or main effects were observed, pairwise comparisons with a Bonferroni correction were applied. The data were reported for each dependent variable as mean ± standard deviation (SD). Statistical significance was set at *p* < 0.05.

## 3. Results

**Figure 2:**
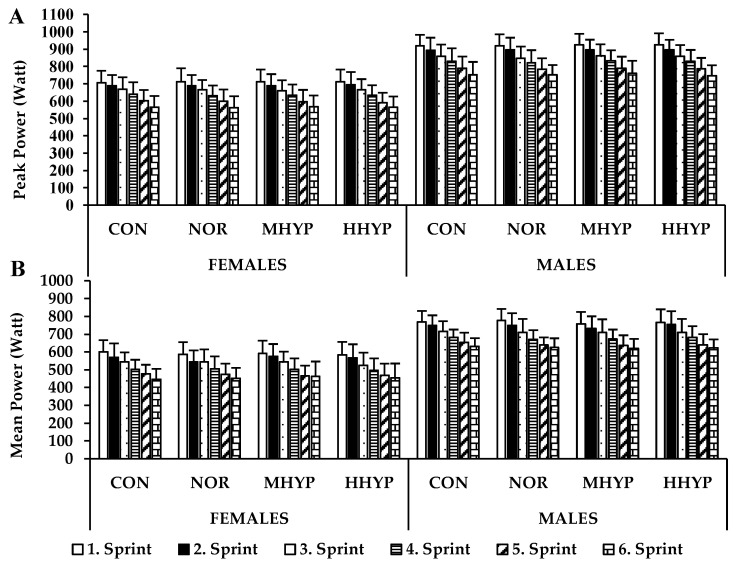
Peak and Mean Power values during 6 × 15 s SIT protocol. CON: at 900 m without wearing hypoxia generator mask; NOR: at 900 m wearing hypoxia generator mask; MHYP: at 2500 m wearing hypoxia generator mask; HHYP: at 3500 m wearing hypoxia generator mask.

The results of the ANOVA showed that there were no conditions × sprints × genders (*p* = 0.98, η_p_^2^ = 0.03), conditions × sprints (*p* = 0.98, η_p_^2^ = 0.03) or conditions × genders (*p* = 0.55, η_p_^2^ = 0.05) interactions in PPO values. There was also no main effect for conditions (*p* = 0.55, η_p_^2^ = 0.05), although a main effect for genders (*p* = 0.01, η_p_^2^ = 0.81) and sprints (*p* = 0.01, η_p_^2^ = 0.96) were found, meaning that males had higher PPO values than females (*p* = 0.01) and sprint performance decreased from the first to the sixth sprint (Figure 2A). Similarly, conditions × sprints × genders (*p* = 0.33, η_p_^2^ = 0.08), conditions × sprints (*p* = 0.42, η_p_^2^ = 0.07) or conditions × genders (*p* = 0.31, η_p_^2^ = 0.09) interaction was not detected in MPO values. Although MPO did not differ between conditions (*p* = 0.44, η_p_^2^ = 0.07), a main effect for genders (*p* = 0.01, η_p_^2^ = 0.84) and sprints (*p* = 0.01, η_p_^2^ = 0.94) were found, with greater MPO values for males and for the first sprint compared to the last sprint (Figure 2B). 

All HRV parameters (SDNN, SDSD, RMSSD, TP, LF, HF and LF/HF) did not change between conditions (*p* > 0.05), or between genders (*p* > 0.05). However, females had higher HF (*p* = 0.03) and TP (*p* = 0.02) values than males. Conditions × times × genders interaction was not significant in all HRV parameters (*p* > 0.05) (Table 1). 

The CP parameter response accuracy did not change between conditions (*p* = 0.92, η_p_^2^ = 0.01), genders (*p* = 0.91, η_p_^2^ = 0.01), times (*p* = 0.47, η_p_^2^ = 0.04), and no conditions × times × genders interaction was found (*p* = 0.19, η_p_^2^ = 0.12) (Table 2). Further, the CP parameter reaction time was not different between conditions (*p* = 0.24, η_p_^2^ = 0.10), genders (*p* = 0.68, η_p_^2^ = 0.01), times (*p* = 0.82, η_p_^2^ = 0.01), and no conditions × times × genders interaction was found (*p* = 0.82, η_p_^2^ = 0.02) (Table 2). However, hypoxia significantly affected lactate levels (*p* = 0.01, η_p_^2^ = 0.36), with post-hoc analysis revealing greater lactate after HHYP compared to CON (*p* = 0.01) and NOR (*p* = 0.01). However, there were no significant differences between HHYP and MHYP (*p* = 0.10) (Table 2). The CP parameter response accuracy did not change between conditions (*p* = 0.92, η_p_^2^ = 0.01), genders (*p* = 0.91, η_p_^2^ = 0.01), times (*p* = 0.47, η_p_^2^ = 0.04), and no conditions × times × genders interaction was found (*p* = 0.19, η_p_^2^ = 0.12) (Table 2). Further, the CP parameter reaction time was not different between conditions (*p* = 0.24, η_p_^2^ = 0.10), genders (*p* = 0.68, η_p_^2^ = 0.01), times (*p* = 0.82, η_p_^2^ = 0.01), and no conditions × times × genders interaction was found (*p* = 0.82, η_p_^2^ = 0.02) (Table 2). However, hypoxia significantly affected lactate levels (*p* = 0.01, η_p_^2^ = 0.36), with post-hoc analysis revealing greater lactate after HHYP compared to CON (*p* = 0.01) and NOR (*p* = 0.01). However, there were no significant differences between HHYP and MHYP (*p* = 0.10) (Table 2). 

The HR values, measured after each 15 s sprint, were not different between conditions (*p* = 0.49, η_p_^2^ = 0.06) and genders (*p* = 0.05, η_p_^2^ = 0.28). However, HR increased from the first to the sixth sprint (*p* = 0.01, η_p_^2^ = 0.99). Conditions × times × genders interaction was not significant (*p* = 0.93, η_p_^2^ = 0.04) (Table 3). The RPE was not different between conditions (*p* = 0.39, η_p_^2^ = 0.07), genders (*p* = 0.29, η_p_^2^ = 0.08), and without conditions × times × genders interaction (*p* = 0.89, η_p_^2^ = 0.04), although increased from the first to the sixth sprint (*p* = 0.01, η_p_^2^ = 0.97) (Table 3). The SpO_2_ measured after each sprint bout was different between conditions (*p* = 0.01, η_p_^2^ = 0.80), with the post-hoc analysis revealing lower values after HHYP compared to CON (*p* = 0.01), NOR (*p* = 0.01; SpO_2_), and MHYP (*p* = 0.01). Further, lower SpO_2_ values were noted after MHYP compared to CON (*p* = 0.01) and NOR (*p* = 0.01), without difference between CON and NOR (*p* = 0.68). Additionally, a conditions × times × genders interaction was found (*p* = 0.01, η_p_^2^ = 0.10), with females having higher SpO_2_ values than males after MHYP and HHYP conditions (*p* < 0.05) (Table 3). 

## 4. Discussion

This study examined the acute effects of different doses of normobaric hypoxia on SIT performance, HRV and CP in amateur-trained team-sport players by comparing potential sex differences. The primary findings show that acute normobaric hypoxia with different doses had no effect on SIT performance, HRV and CP between female and male team sports players. However, lower SpO_2_ values were observed in HHYP compared to MHYP conditions and females had higher SpO_2_ values than males after MHYP and HHYP conditions. Collectively, these findings conflict with our experimental hypothesis and reveal that the acute mild or high dose normobaric hypoxia does not influence SIT performance, HRV and CP in female and male team sports players.

The acute MHYP and HHYP did not provide any difference in PPO and MPO during the SIT protocol compared to normoxia. These observations are consistent with some [15,40], but not all [41,42,43] previous studies. These discrepancy findings between the studies are likely due to the application of considerably different exercise modalities and exercise protocols regarding work-to-rest ratio (e.g., durations of sprint and/or recovery). For example, whilst Brocherie et al. [40] reported a decrease in repeated sprint performance during 6 × 15 s sprints separated with 30 sec recoveries, in hypoxia compared to normoxia, Kon et al. [42] reported no changes in sprint performance during a 4 × 30 s sprint with a 4 min rest. Further, it has been suggested that sprint performance decreases during repeated sprints in hypoxia when the recovery is less than 30 s [40,41]. Together, our findings combined with previous observations reveal that when the recovery periods between sprints are sufficient (2–5 min), the sprint performance will not deteriorate as the energy stores, such as muscle phosphocreatine (PCr), would recover completely although hypoxia can increase non-oxidative glycolysis [40,41]. The present study showed that PPO and MPO were greater in males than females, which is in accordance with others who illustrated that differences between males and females may be attributed to body composition differences [44,45,46]. To the best of our knowledge, this is the first study to focus on sex-based performance differences across SIT under different doses of acute normobaric hypoxia. Previously, only one study assessed the sex-based differences in repeated-sprint performance but performed under only one hypoxic condition (F_i_O_2_: 13%) [47]. That study reported that sprint performance decreased in hypoxic conditions compared to normoxic conditions, but no differences were found between sexes. Our findings are in line with the study by Smith and Billaut [47] regarding sex-based differences, but inconsistent with regard to sprint performance.

It has been reported that in a hypoxic environment, tissue saturation index and deoxyhemoglobin decrease continuously as the dose of hypoxia increases [48], and physiological changes such as cerebral deoxygenation may have deleterious effects on CP [49]. Surprisingly, our study found no difference in CP between hypoxic conditions. These findings may indicate that the duration of hypoxic exposure was too short due to the low exercise volume and this situation does not provide sufficient physiological stress on the central nervous system. Moreover, our rest interval between sprints (2 min) may have provided adequate time for brain oxygenation, preventing deterioration of cognitive function.

Our findings have shown that HRV variables were not affected by hypoxia. Most studies examining the effect of hypoxia on HRV have been conducted in low-intensity physical activities. In one study, it has been reported that low-frequency component (LF) and LF/high-frequency component (HF) values decrease more after exercise in hypoxia (between 1200–3000 m) compared to normoxia [50]. Another study found that HR recovery decreases after a submaximal workload (5 min) at 2400 m of altitude [51]. Aras and Coskun [52] examined the effect of a single bout of the Wingate test on HRV variables under various levels of altitude (162 m, 1015 m, 2146 m, and 3085 m) in healthy males and females, and reported no changes in HRV variables at any altitudes. However, Botek et al. [53] found significantly decreased vagal activity after exposure to hypoxia at 6200 m in healthy males. The most obvious explanation for the inconsistent findings is because the extreme altitude was used by Botek et al. [53], which is not applicable and practical for at least team-sport athletes. The present study also found no differences between sexes regarding HRV. Those findings are in accordance with previous studies, which showed a lack of differences between sexes related to the cardiac system response to hypoxia [54,55].

We observed higher BLa concentration in HHYP compared to CON and NOR. As the altitude increases, the increase in BLa concentration with the decrease in inhaled oxygen indicates that non-oxidative glycolysis increases [15]. Our findings are consistent with previous studies reporting higher BLa concentration during (I) 3 sets of 6 × 10 s sprints at different doses of hypoxia (FiO_2_: 14.5%, 13.5% and 12.5%) compared to normoxia [17] and (II) during 4 sets of 4 × 4 s sprints in hypoxic condition (FiO_2_: 14%) compared to in normoxic condition [56]. There was a dose-dependent lowering in SpO_2_ in the current study as the FiO_2_ of the applied gas mixture was decreased. This finding is in line with previous studies reporting greater decreases in SpO_2_ during hypoxic exercise [57] and a dose-dependent decrease in muscle oxygenation [58]. This decrease in SpO_2_ increases the stress on glycolytic flux and therefore may stimulate the upregulation of this energy pathway (anaerobic) [59].

We acknowledge some potential limitations in our study. Firstly, we did not assess the female menstrual cycle, which might have affected some of our findings. Nonetheless, all testing protocols were completed in 7–8 days, reducing the chances that female participants went through different menstrual phases during this short period of time. Secondly, the assessment of respiratory gases (e.g., oxygen consumption and carbon dioxide production) and ventilation parameters (e.g., tidal volume or minute ventilation) might be helpful parameters to better understand the responses of the cardiac system to different doses of acute hypoxia.

## 5. Conclusions

In conclusion, these findings suggest that different doses of acute normobaric hypoxia had no effect on SIT performance, HRV and CP and highlight that there are no sex-based differences in these acute responses in normobaric hypoxia. These observations do not support acute normobaric hypoxia, at least less than 12 min of hypoxia, to alter SIT performance in amateur-trained male and female team-sports players.

## Figures and Tables

**Table 1:** Heart rate variability values measured before and after SIT protocol.

	Pre SIT	Post SIT	Pre SIT	Post SIT
	M (SD)	M (SD)	M (SD)	M (SD)
	Females		Males	
SDNN				
CON	80.4 (43.3)	29.6 (16.9)	83.7 (24.5)	20.8 (11.5)
NOR	74.7 (42.4)	26.3 (8.9)	90.2 (28.3)	23.7 (13.9)
MHYP	83.7 (45.8)	26.3 (20.4)	99.8 (69.5)	26.8 (24.2)
HHYP	89.2 (53.9)	28.5 (26.1)	92.7 (68.3)	29.1 (23.9)
SDSD				
CON	116.0 (71.9)	21.9 (12.7)	93.0 (21.0)	16.1 (14.1)
NOR	126.8 (97.1)	17.2 (5.7)	108.2 (39.3)	14.5 (8.7)
MHYP	137.5 (97.5)	23.2 (13.1)	116.8 (51.5)	18.0 (11.7)
HHYP	135.2 (90.8)	24.4 (15.7)	111.7 (46.2)	19.0 (10.0)
RMSSD				
CON	95.8 (65.6)	23.5 (14.5)	74.2 (16.5)	14.6 (12.8)
NOR	98.1 (76.3)	15.9 (8.5)	79.9 (22.8)	15.6 (10.2)
MHYP	114.9 (82.1)	24.9 (16.3)	97.2 (49.3)	14.3 (12.9)
HHYP	128.2 (100.7)	23.6 (12.2)	105.0 (49.9)	19.0 (16.2)
TP				
CON	2963.9 (1647.3)	248.5 (140.9)	2180.9 (1259.1)	170.3 (239.4)
NOR	2882.2 (1751.1)	230.4 (80.9)	2446.2 (1018.8)	163.3 (234.1)
MHYP	3326.8 (1532.9)	244.3 (140.7)	2401.8 (874.4)	176.4 (250.5)
HHYP	3286.2 (1461.4)	271.6 (164.7)	2542.3 (692.9)	145.2 (121.1)
LF				
CON	859.8 (363.3)	143.8 (99.3)	1105.3 (993.0)	80.8 (161.3)
NOR	865.0 (456.6)	122.8 (79.2)	1230.3 (868.9)	102.6 (164.7)
MHYP	1168.2 (691.5)	140.9 (106.5)	1242.0 (748.4)	79.6 (101.4)
HHYP	1161.5 (728.8)	163.0 (128.7)	1213.0 (661.0)	84.2 (62.5)
HF				
CON	1740.8 (1447.0)	87.6 (58.1)	849.7 (452.3)	62.8 (109.7)
NOR	1636.0 (1374.2)	113.8 (58.2)	1033.3 (517.1)	106.1 (159.6)
MHYP	1701.0 (1000.0)	86.6 (75.4)	1239.0 (554.0)	151.1 (197.0)
HHYP	1784.3 (1136.0)	98.2 (95.7)	1398.4 (688.9)	131.3 (140.7)
LF/HF				
CON	0.8 (0.4)	5.5 (1.8)	1.4 (1.7)	5.5 (5.0)
NOR	1.3 (0.7)	5.3 (1.8)	1.8 (1.8)	5.1 (5.2)
MHYP	0.9 (0.4)	5.2 (2.6)	1.4 (1.0)	4.0 (4.2)
HHYP	0.9 (0.4)	5.3 (2.7)	1.4 (1.1)	3.2 (3.3)

Pre SIT: before SIT protocol; Post SIT: immediately after SIT protocol. M (SD): Mean, standart deviation

**Table 2:** Cognitive function and lactate parameters measured before and after SIT protocol.

	Pre SIT	Post SIT	Pre SIT	Post SIT
	M (SD)	M (SD)	M (SD)	M (SD)
	Females		Males	
Response Accuracy (%)				
CON	92.2 (2.6)	92.0 (2.7)	93.1 (2.7)	93.4 (2.0)
NOR	92.6 (2.6)	92.6 (2.5)	92.9 (1.8)	92.9 (2.2)
MHYP	92.8 (2.8)	92.9 (2.6)	93.2 (3.1)	92.2 (2.5)
HHYP	93.1 (2.6)	93.8 (2.0)	93.3 (1.7)	91.6 (1.8)
Reaction Time (ms)				
CON	525.2 (24.2)	522.4 (23.7)	516.1 (24.3)	520.3 (17.5)
NOR	545.8 (41.3)	527.3 (32.5)	529.2 (25.4)	522.3 (32.9)
MHYP	531.5 (44.3)	532.4 (20.4)	529.2 (26.7)	536.2 (35.1)
HHYP	520.4 (20.4)	535.4 (20.1)	528.3 (19.5)	537.7 (30.6)
Lactate (mmol)				
CON	1.1 (0.1)	10.2 (1.1)	1.0 (0.2)	11.8 (2.1)
NOR	1.1 (0.2)	10.2 (1.4)	1.0 (0.1)	11.9 (2.1)
MHYP	1.0 (0.2)	11.1 (1.9)	1.1 (0.2)	12.3 (1.7)
HHYP	1.0 (0.1)	11.6 (1.9) *	1.0 (0.2)	13.3 (1.6) *

M (SD): Mean, standart deviation; *: Significantly different than CON and NOR

**Table 3:** Heart rate, ratings of perceived exertion and SpO_2_ values measured after each sprint.

	Females				Males			
	Heart Rate							
	CON	NOR	MHYP	HHYP	CON	NOR	MHYP	HHYP
Sprint 1	162.1 (9.0)	163.6 (8.3)	164.3 (4.8)	164.3 (6.0)	165.3 (6.8)	166.0 (5.5)	165.3 (4.6)	166.4 (4.5)
Sprint 2	168.0 (5.7)	165.6 (8.2)	166.9 (8.5)	166.3 (7.7)	167.6 (8.0)	168.5 (7.0)	167.4 (5.1)	167.2 (4.7)
Sprint 3	171.1 (7.8)	170.3 (6.3)	168.9 (8.0)	168.2 (3.8)	173.3 (7.3)	172.3 (8.7)	170.6 (6.0)	169.3 (5.7)
Sprint 4	170.2 (7.2)	172.4 (6.8)	171.2 (7.8)	169.3 (8.7)	176.0 (7.0)	176.5 (6.6)	174.4 (5.1)	172.5 (6.1)
Sprint 5	171.4 (7.3)	172.7 (7.9)	170.2 (7.9)	171.1 (7.0)	179.2 (7.7)	178.6 (6.7)	179.3 (6.9)	177.4 (8.5)
Sprint 6	170.9 (6.3)	173.7 (6.5)	171.0 (7.6)	173.6 (4.8)	182.9 (6.5)	182.3 (7.6)	180.9 (8.0)	180.3 (8.2)
	Ratings of perceived exertion							
Sprint 1	14.4 (1.3)	14.6 (0.7)	14.6 (1.1)	14.9 (1.3)	13.3 (1.8)	14.0 (1.8)	13.4 (1.8)	13.6 (1.8)
Sprint 2	15.7 (1.4)	15.4 (1.5)	15.5 (1.4)	15.6 (1.5)	14.7 (1.2)	15.3 (2.0)	14.3 (2.7)	15.0 (19)
Sprint 3	16.3 (1.6)	16.3 (1.3)	16.8 (1.2)	16.4 (1.1)	15.5 (1.9)	16.3 (2.3)	15.6 (2.1)	16.8 (2.2)
Sprint 4	17.6 (1.7)	17.0 (1.5)	17.5 (1.6)	17.3 (1.7)	17.3 (1.7)	17.7 (1.7)	16.7 (2.1)	17.7 (2.0)
Sprint 5	18.3 (1.5)	18.1 (1.2)	18.4 (1.5)	18.3 (1.5)	18.1 (1.5)	18.3 (1.2)	17.6 (1.9)	18.3 (1.6)
Sprint 6	19.0 (1.0)	18.5 (1.4)	19.0 (1.1)	18.6 (1.3)	18.6 (1.8)	18.8 (1.4)	18.1 (1.6)	18.9 (1.3)
SpO_2_								
Sprint 1	90.9 (1.7)	90.4 (1.1)	87.3 (2.1)	85.6 (2.6)	90.3 (1.7)	90.3 (1.5)	87.6 (2.2)	87.5 (2.0)
Sprint 2	90.4 (1.8)	90.2 (1.3)	87.6 (1.3)	85.1 (1.8)	90.3 (1.9)	90.1 (2.7)	86.4 (1.5)	85.6 (1.7)
Sprint 3	90.0 (2.1)	89.6 (1.8)	87.2 (1.0)	85.0 (1.6)	89.8 (2.3)	90.1 (2.6)	86.0 (1.8)	84.3 (1,9)
Sprint 4	89.8 (2.1)	89.1 (1.9)	86.8 (1.2)	84.8 (1.7)	89.6 (2.3)	89.3 (2.9)	85.5 (2.2)	83.7 (1.6)
Sprint 5	89.6 (2.1)	89.2 (2.2)	86.3 (1.6)	84.4 (2.0)	89.4 (2.6)	89.0 (2.7)	84.7 (1.8)	83.3 (1.4)
Sprint 6	89.6 (2.1)	89.3 (1.3)	86.1 (1.3)	84.0 (2.1)	89.4 (2.5)	88.7 (2.6)	84.3 (2.3)	82.8 (1.5)

## Data Availability

The data presented in this study are available on request from the corresponding author. The data are not publicly available due to restrictions privacy.

## References

[1] Yamagishi T., Babraj J. (2017). Effects of reduced-volume of sprint interval training and the time course of physiological and performance adaptations. Scand. J. Med. Sci. Sport..

[2] Burgomaster K.A., Hughes S.C., Heigenhauser G.J., Bradwell S.N., Gibala M.J. (2005). Six sessions of sprint interval training increases muscle oxidative potential and cycle endurance capacity in humans. J. Appl. Physiol..

[3] Clark S.A., Chen Z.-P., Murphy K.T., Aughey R., McKenna M., Kemp B.E., Hawley J.A. (2004). Intensified exercise training does not alter AMPK signaling in human skeletal muscle. Am. J. Physiol. Endocrinol. Metab..

[4] Burgomaster K.A., Heigenhauser G.J., Gibala M.J. (2006). Effect of short-term sprint interval training on human skeletal muscle carbohydrate metabolism during exercise and time-trial performance. J. Appl. Physiol..

[5] Edge J., Bishop D., Goodman C. (2006). Effects of chronic NaHCO_3_ ingestion during interval training on changes to muscle buffer capacity, metabolism, and short-term endurance performance. J. Appl. Physiol..

[6] Vogt M., Puntschart A., Geiser J., Zuleger C., Billeter R., Hoppeler H. (2001). Molecular adaptations in human skeletal muscle to endurance training under simulated hypoxic conditions. J. Appl. Physiol..

[7] Schmutz S., Däpp C., Wittwer M., Durieux A.C., Mueller M., Weinstein F., Vogt M., Hoppeler H., Flück M. (2010). A hypoxia complement differentiates the muscle response to endurance exercise. Exp. Physiol..

[8] Zoll J., Ponsot E., Dufour S., Doutreleau S., Ventura-Clapier R., Vogt M., Hoppeler H., Richard R., Flück M. (2006). Exercise training in normobaric hypoxia in endurance runners. III. Muscular adjustments of selected gene transcripts. J. Appl. Physiol..

[9] Puype J., Van Proeyen K., Raymackers J.-M., Deldicque L., Hespel P. (2013). Sprint interval training in hypoxia stimulates glycolytic enzyme activity. Med. Sci. Sport. Exerc..

[10] Faiss R., Léger B., Vesin J.-M., Fournier P.-E., Eggel Y., Dériaz O., Millet G.P. (2013). Significant molecular and systemic adaptations after repeated sprint training in hypoxia. PLoS ONE.

[11] Warnier G., Benoit N., Naslain D., Lambrecht S., Francaux M., Deldicque L. (2020). Effects of sprint interval training at different altitudes on cycling performance at sea-level. Sports.

[12] Millet G.P., Girard O., Beard A., Brocherie F. (2019). Repeated sprint training in hypoxia–an innovative method. Dtsch. Z. Für Sportmed..

[13] Faiss R., Girard O., Millet G.P. (2013). Advancing hypoxic training in team sports: From intermittent hypoxic training to repeated sprint training in hypoxia. Br. J. Sport Med..

[14] Millet G.P., Faiss R. (2012). Hypoxic conditions and exercise-to-rest ratio are likely paramount. Sports Med..

[15] Bowtell J.L., Cooke K., Turner R., Mileva K.N., Sumners D.P. (2014). Acute physiological and performance responses to repeated sprints in varying degrees of hypoxia. J. Sci. Med. Sport.

[16] Goods P.S., Dawson B., Landers G.J., Gore C.J., Peeling P. (2015). No additional benefit of repeat-sprint training in hypoxia than in normoxia on sea-level repeat-sprint ability. J. Sport. Sci. Med..

[17] Khaosanit P., Hamlin M.J., Graham K.S., Boonrod W. (2018). Acute effect of different normobaric hypoxic conditions on shuttle repeated sprint performance in futsal players. J. Phys. Educ. Sport.

[18] Kon M., Nakagaki K., Ebi Y., Nishiyama T., Russell A.P. (2015). Hormonal and metabolic responses to repeated cycling sprints under different hypoxic conditions. Growth Horm. IGF Res..

[19] Karabiyik H., Eser M.C., Guler O., Yasli B.C., Ertetik G., Sisman A., Koz M., Gabrys T., Pilis K., Karayigit R. (2021). The effects of 15 or 30 s SIT in normobaric hypoxia on aerobic, anaerobic performance and critical power. Int. J. Environ. Res. Public Health.

[20] Zupet P., Princi T., Finderle Z. (2009). Effect of hypobaric hypoxia on heart rate variability during exercise: A pilot field study. Eur. J. Appl. Physiol..

[21] Yamamoto Y., Hoshikawa Y., Miyashita M. (1996). Effects of acute exposure to simulated altitude on heart rate variability during exercise. J. Appl. Physiol..

[22] Buchheit M., Simon C., Piquard F., Ehrhart J., Brandenberger G. (2004). Effects of increased training load on vagal-related indexes of heart rate variability: A novel sleep approach. Am. J. Physiol. Heart Circ. Physiol..

[23] Petrassi F.A., Hodkinson P.D., Walters P.L., Gaydos S.J. (2012). Hypoxic hypoxia at moderate altitudes: Review of the state of the science. Aviat. Space Environ. Med..

[24] Komiyama T., Katayama K., Sudo M., Ishida K., Higaki Y., Ando S. (2017). Cognitive function during exercise under severe hypoxia. Sci. Rep..

[25] Galvin H.M., Cooke K., Sumners D.P., Mileva K.N., Bowtell J.L. (2013). Repeated sprint training in normobaric hypoxia. Br. J. Sport. Med..

[26] Tian Z., Kim B.-Y., Bae M.-J. (2020). A study on the effect of wearing masks on stress response. Memory.

[27] Sandoval D.A., Matt K.S. (2003). Effects of the oral contraceptive pill cycle on physiological responses to hypoxic exercise. High Alt. Med. Biol..

[28] Laurent C.M., Green J.M., Bishop P.A., Sjökvist J., Schumacker R.E., Richardson M.T., Curtner-Smith M. (2010). Effect of gender on fatigue and recovery following maximal intensity repeated sprint performance. J. Sport. Med. Phys. Fit..

[29] Laurent C.M., Vervaecke L.S., Kutz M.R., Green J.M. (2014). Sex-specific responses to self-paced, high-intensity interval training with variable recovery periods. J. Strength Cond. Res..

[30] Sandoval D.A., Matt K.S. (2002). Gender differences in the endocrine and metabolic responses to hypoxic exercise. J. Appl. Physiol..

[31] Esbjornsson-Liljedahl M., Bodin K., Jansson E. (2002). Smaller muscle ATP reduction in women than in men by repeated bouts of sprint exercise. J. Appl. Physiol..

[32] Storer T.W., Davis J.A., Caiozzo V.J. (1990). Accurate prediction of VO2max in cycle ergometry. Med. Sci. Sport. Exerc..

[33] Sims S.T., Ware L., Capodilupo E.R. (2021). Patterns of endogenous and exogeneous ovarian hormone modulation on recovery metrics across the menstrual cycle. BMJ Open Sport Exerc. Med..

[34] Sims S.T., Heather A.K. (2018). Myths and methodologies: Reducing scientific design ambiguity in studies comparing sexes and/or menstrual cycle phases. Exp. Physiol..

[35] Heffernan K.S., Kelly E.E., Collier S.R., Fernhall B. (2006). Cardiac autonomic modulation during recovery from acute endurance versus resistance exercise. Eur. J. Cardiovasc. Prev. Rehabil..

[36] Peçanha T., Bartels R., Brito L.C., Paula-Ribeiro M., Oliveira R.S., Goldberger J.J. (2017). Methods of assessment of the post-exercise cardiac autonomic recovery: A methodological review. Int. J. Cardiol..

[37] Karayigit R., Naderi A., Akca F., Cruz C.J.G.D., Sarshin A., Yasli B.C., Ersoz G., Kaviani M. (2020). Effects of Different Doses of Caffeinated Coffee on Muscular Endurance, Cognitive Performance, and Cardiac Autonomic Modulation in Caffeine Naive Female Athletes. Nutrients.

[38] Eriksen B.A., Eriksen C.W. (1974). Effects of noise letters upon the identification of a target letter in a nonsearch task. Percept. Psychophys..

[39] Cohen J. (1992). A power primer. Psych. Bull..

[40] Brocherie F., Girard O., Faiss R., Millet G.P. (2017). Effects of repeated-sprint training in hypoxia on sea-level performance: A meta-analysis. Sport. Med..

[41] Girard O., Brocherie F., Millet G.P. (2017). Effects of altitude/hypoxia on single-and multiple- sprint performance: A comprehensive review. Sport. Med..

[42] Kon M., Ohiwa N., Honda A., Matsubayashi T., Ikeda T., Akimoto T., Suzuki T., Hirano Y., Rusell A.P. (2015). Effects of systemic hypoxia on human muscular adaptations to resistance exercise training. Physiol. Rep..

[43] Ogawa T., Hayashi K., Ichinose M., Wada H., Nishiyasu T. (2007). Metabolic response during intermittent graded sprint running in moderate hypobaric hypoxia in competitive middle- distance runners. Eur. J. Appl. Physiol..

[44] Freese E.C., Gist N.H., Cureton K.J. (2013). Physiological responses to an acute bout of sprint interval cycling. J. Strength Cond. Res..

[45] Murphy M.M., Patton J.F., Frederick F.A. (1986). Comparative anaerobic power of men and women. Aviat. Space Environ. Med..

[46] Magal M., Liette N.C., Crowley S.K., Hoffman J.R., Thomas K.S. (2021). Sex-based performance responses to an acute sprint interbal cycling training session in collegiate athletes. Res. Q. Exerc. Sport.

[47] Smith K.J., Billaut F. (2012). Tissue oxygenation in men and women during repeated-sprint exercise. Int. J. Sport. Physiol. Perform..

[48] Willis S.J., Alvarez L., Millet G.P., Borrani F. (2017). Changes in muscle and cerebral deoxygenation and perfusion during repeated sprints in hypoxia to exhaustion. Front. Physiol..

[49] Ochi G., Yamada Y., Hyodo K., Suwabe K., Fukuie T., Byun K., Dan I., Soya H. (2018). Neural basis for reduced executive performance with hypoxic exercise. Neuroimage.

[50] Povea C., Schmitt L., Brugniaux J., Nicolet G., Richalet J.-P., Fouillot J.-P. (2005). Effects of intermittent hypoxia on heart rate variability during rest and exercise. High Alt. Med. Biol..

[51] Al Haddad H., Mendez-Villanueva A., Bourdon P.C., Buchheit M. (2012). Effect of acute hypoxia on post-exercise parasympathetic reactivation in healthy men. Front. Physiol..

[52] Aras D., Coskun B. (2016). The changes on the HRV after a Wingate anaerobic test in different simulated altitudes in healthy, physically-active adults. Acta Med. Mediterr..

[53] Botek M., Krejčí J., De Smet S., Gába A., McKune A.J. (2015). Heart rate variability and arterial oxygen saturation response during extreme normobaric hypoxia. Auton. Neurosci..

[54] Boss C.J., Mellor A., O’Hara J.P., Tsakirides C., Woods D.R. (2016). The effects of sex on cardiopulmonary responses to acute normobaric hypoxia. High Alt. Med. Biol..

[55] Burtscher M., Philadelphy M., Gatterer H., Burtscher J., Faulbaher M., Nachbauer W., Likar R. (2019). Physiological responses in humans acutely exposed to high altitude (3480 m): Minute ventilation and oxygenation are predictive for the development of acute mountain sickness. High Alt. Med. Biol..

[56] Morrison J., McLellan C., Minahan C. (2015). A clustered repeated-sprint running protocol for team-sport athletes performed in normobaric hypoxia. J. Sport. Sci. Med..

[57] Calbet J., Boushel R., Rådegran G., Søndergaard H., Wagner P.D., Saltin B. (2003). Determinants of maximal oxygen uptake in severe acute hypoxia. Am. J. Physiol. Regul. Integr. Comp. Physiol..

[58] Goodall S., Ross E.Z., Romer L.M. (2010). Effect of graded hypoxia on supraspinal contributions to fatigue with unilateral knee-extensor contractions. J. Appl. Physiol..

[59] Faiss R., Rapillard A. (2020). Repeated Sprint Training in Hypoxia: Case Report of Performance Benefits in a Professional Cyclist. Front. Sport. Act. Living.

